# Weather Extremes Shock Maize Production: Current Approaches and Future Research Directions in Africa

**DOI:** 10.3390/plants13121585

**Published:** 2024-06-07

**Authors:** Shaolong Du, Wei Xiong

**Affiliations:** 1 College of Agronomy, Henan Agricultural University, Zhengzhou 450046, China; mariomalone@163.com; 2 International Maize and Wheat Improvement Center, Zhengzhou 450046, China

**Keywords:** climate change, adaptation, gaps, effect, yield, food security, resilience, modeling

## Abstract

Extreme weather events have led to widespread yield losses and significant global economic damage in recent decades. African agriculture is particularly vulnerable due to its harsh environments and limited adaptation capacity. This systematic review analyzes 96 articles from Web of Science, Science Direct, and Google Scholar, focusing on biophysical studies related to maize in Africa and worldwide. We investigated the observed and projected extreme weather events in Africa, their impacts on maize production, and the approaches used to assess these effects. Our analysis reveals that drought, heatwaves, and floods are major threats to African maize production, impacting yields, suitable cultivation areas, and farmers’ livelihoods. While studies have employed various methods, including field experiments, statistical models, and process-based modeling, African research is often limited by data gaps and technological constraints. We identify three main gaps: (i) lack of reliable long-term experimental and empirical data, (ii) limited access to advanced climate change adaptation technologies, and (iii) insufficient knowledge about specific extreme weather patterns and their interactions with management regimes. This review highlights the urgent need for targeted research in Africa to improve understanding of extreme weather impacts and formulate effective adaptation strategies. We advocate for focused research on data collection, technology transfer, and integration of local knowledge with new technologies to bolster maize resilience and food security in Africa.

## 1. Introduction

The world will need to increase food production by 60% to feed an estimated nine billion people by 2050 [1]. This challenge will be particularly daunting in Africa, which faces the most rapid population growth, increasing water and land scarcity, shifting consumption patterns, and limited access to resources and technology [2]. Climate change poses additional obstacles to this endeavor. Africa is considered as the continent most vulnerable to climate variability and change [3]. Climate change impacts are becoming more intense and frequent than ever, as the observed impacts of extreme climate events have intensified in recent years, including prolonged dry spells, abnormal rainfall patterns, consequent water shortages, and heat stress [4,5,6]. Because of the large dependence of key African livelihoods on climate, particularly rainfall [7], understanding the changes in climate extremes under climate change and their influences on crop production is paramount and urgent to enhance the adaptation towards climate change in Africa.

Maize is one of the most widely cultivated staple crops and one of the few crops profoundly affecting people’s livelihoods in Africa [8]. Maize is cultivated on over 38 million hectares in sub-Saharan Africa (SSA), which accounted for 35% of the total cereal area and 46% of cereal production between 2010 and 2020 [9]. More than half of the countries in SSA allocate over 50% of their cereal area to maize production [10]. Maize is the most important source of dietary protein and the second most important source of calories in SSA, accounting for almost half the calories and protein consumed in Eastern and Southern Africa (ESA) and one-fifth of the calories and protein consumed in West Africa [11]. Despite the importance of maize in SSA, current production is insufficient in most countries, and yields remain among the lowest in the world because of an array of biophysical and socioeconomic constraints [12].

Due to the heavy reliance on rain-fed agriculture, maize harvests in SSA can be severely reduced by extreme weather events, such as heatwaves, droughts, and floods. For example, drought led to a decrease in maize production in SSA in 2015/16, necessitating humanitarian assistance [13]; flooding in 2022 destroyed 60% of planted maize in South Africa [14]. Limited access to technology and inputs further contributed to the vulnerability of maize production to weather extremes [15]. Because of its economic importance and severe production loss from climatic disasters, maize has been one of the most studied crops in relation to the impacts of climate change, climate variability, and extreme weather phenomena. Varying methods have been used to examine the impacts. For example, agrometeorological indices were examined to detect changes in climate extremes and their implications on maize production [16]. Experimental data were used to understand how maize yield responds to various conditions and perturbations [17]. Statistical models fitting local, regional, or national yields to weather variables have been used to deduce crop-climate relationships [18]. Crop modeling, suitability modeling, and machine learning modeling were employed to elucidate observed or projected climate change influences on maize yield, suitability, productivity, or trade [19,20].

Several previous studies have reviewed extreme weather events and their impacts on crops, typically focusing on one or more major crops and extremes, such as Rötter et al. [21], Cogato et al. [22], and Ayanlade et al. [7], as well as the IPCC AR6 [23]. Nevertheless, since most reviews have analyzed multiple crops or focused globally, there remains a deficiency in providing a comprehensive overview of the effects on African maize due to climate change, variability, and extreme weather events. Additionally, given the substantial variations in maize cultivation (i.e., smallholder, low-input) and planting environments (rain-fed) between African nations and those in other continents, failing to account for these distinctions in a global comparison may introduce biases in the conclusions drawn concerning the anticipated impacts of weather extremes.

With this objective in mind, this review synthesizes and analyzes recent research (since 2010) on the effects of climate change, variability, and various weather extremes (drought, heat, heavy rains/hail and storms, flooding, frost, and notably, combinations) on maize production in Africa. The review begins with an outline of the systematic review methodology. Section 3 provides an overview of methods used to measure the impacts of extremes on food production, drawing on global studies and international studies. Section 4 summarizes observed and projected impacts of weather extremes (drought, heat, wetness, and combinations) on maize production, focusing on the effects on yields, suitability areas, production, farmers’ livelihood, and food security. Section 5 discusses adaptation options implemented or proposed in Africa to mitigate the harmful effects of climate change and extreme events, and strategies for enhancing maize resilience. Finally, the paper summarizes our current understanding, identifies knowledge gaps, and recommends areas for future research.

## 2. Methodology

### 2.1. Review Method

Conducting a systematic literature review is an exceptionally effective approach to evaluate a theory or evidence in a specific domain or to assess the accuracy and validity of a particular theory [24]. The review guidelines proposed by Kitchenham and Charters [25] are deemed appropriate for this systematic literature review, as they promote objectivity and transparency. Guided by the review guidelines, the research questions were formulated as the initial step. The review was carried out in alignment with the Preferred Reporting Items for Systematic Reviews and Meta-Analysis (PRISMA) statement [26]. Three databases, namely ScienceDirect, Web of Science, and Google Scholar, were utilized to select relevant research articles. The retrieved research articles were assessed and filtered based on predefined quality criteria. The complete checklist of PRISMA (prisma-statement.org) was employed to conduct and report the findings of this review. This paper aims to provide a comprehensive, state-of-the-art review of the literature concerning weather extremes and their impacts on African agriculture, with a particular focus on maize. The specific objectives of this work are to (i) describe the publication trends over the years, (ii) to summarize the current understanding regarding the observed and anticipated trends of weather extremes and their implications on maize cultivation, and (iii) to identify significant research gaps related to extreme weather events in African agriculture, both in topics and geographical coverage.

### 2.2. Research Questions

The study aims to address the following research questions to guide the systematic review:

Q1: What are the observed and projected trends of climate and weather extremes in Africa?

This question helps us to understand the past and future climate and weather extremes in Africa.

Q2: What are the impacts of climate change and weather extremes on African maize production? And how significant are they compared to the impacts on other continents?

With various methodologies, this question helps us understand the current impacts and magnitude of climate and weather’s extreme effects on Africa’s maize production. This question explores the impacts including yield, cultivation areas, interactions with soil and the environment, pests and diseases, and teleconnection between regions.

Q3: What methods have been used around the globe and in Africa to investigate the impacts of weather extremes on maize?

As tools and technology progress rapidly, answering this question allows us to form a complete picture of the methods used to quantify the effects of weather extremes. These methods are not limited to studies in Africa but also to studies that were conducted in other countries. It also compares each technique’s pros and cons, deducing an appropriate analysis approach for Africa to investigate the impacts of the extreme event with the now available data.

Q4: What adaptation strategies have been implemented, proposed, or assessed in Africa to mitigate the impacts of climate change and weather extremes?

This question enables us to learn about the various adaptation strategies influencing the resilience of maize production.

Q5: What are the research gaps and shortages in Africa, compared to the studies around the globe or on other continents?

This question expands our understanding of the limitations and challenges in existing approaches, which can facilitate our development of case studies in Africa to improve the scale of climate-smart agriculture.

### 2.3. Procedure for Article Search

The systematic review was conducted in three steps: searching, selecting, and analyzing scientific studies to provide evidence related to the impacts of extreme weather events on maize production in Africa, with a focus on climate change and food security. The search strategy was designed based on the research questions and the aim of the systematic literature review. Narrowing the focus from broader concepts to the review’s central idea helped create an effective search strategy. Using search terms, such as “climate change” or “weather extremes”, alone would have generated many published articles from various unrelated fields, complicating the search and deviating from the aim of the review. Redefining the search strategy to include “Africa” AND “maize” AND “adaptation” can reduce the probability of deviating from the scope of the review. The search strings initially retrieved the articles from three databases: ScienceDirect, Web of Science, and Google Scholar. The study included articles published between 2010 and 2023 (up to December 2023).

### 2.4. Article Selection Criteria

The retrieved articles were initially selected based on certain criteria, such as the quality of a journal, the methods used in the study, and the variables investigated (e.g., maize yield, area, quality, heat, drought, compound extremes). Analyzing the abstracts of articles helped identify relevant keywords and select the appropriate articles. Articles were excluded based on the following criteria:

- Articles that addressed maize but did not focus on climate change impacts and adaptation;
- Articles that did not focus on Africa, but instead covered other regions, such as the U.S. or Europe;
- Publications that were not openly accessible;
- Articles published before 2010;
- Articles not published in English.

## 3. Review Results

### 3.1. Article Selected

**Figure 1:**
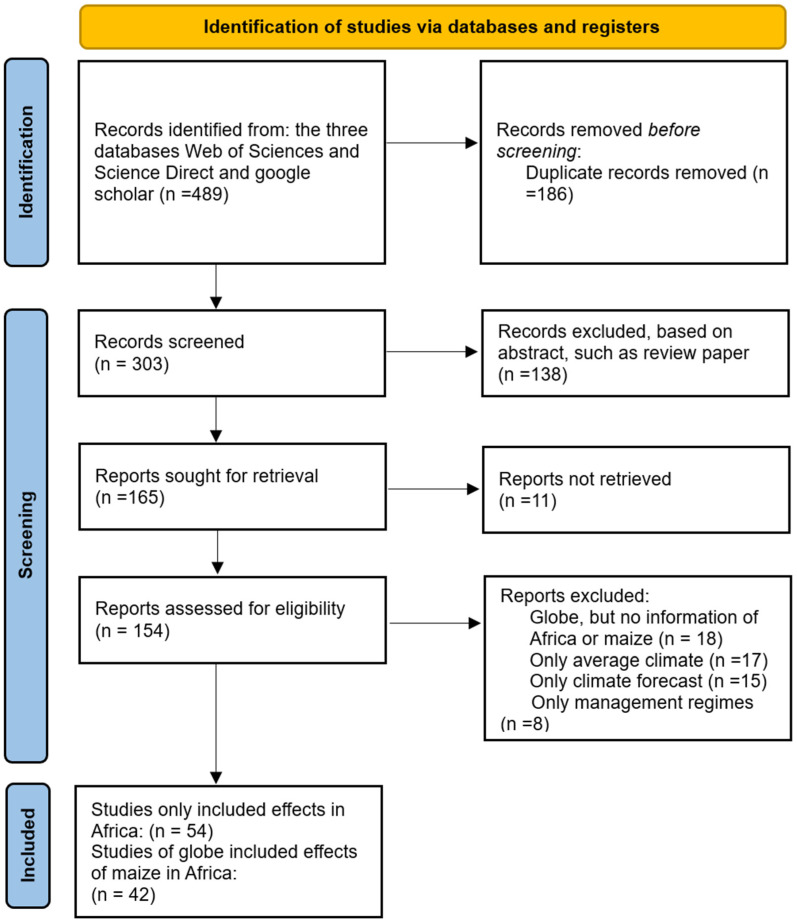
PRISMA flowchart showing the process and criteria applied to the search process. The number of records identified in each step is also reported.

**Figure 2:**
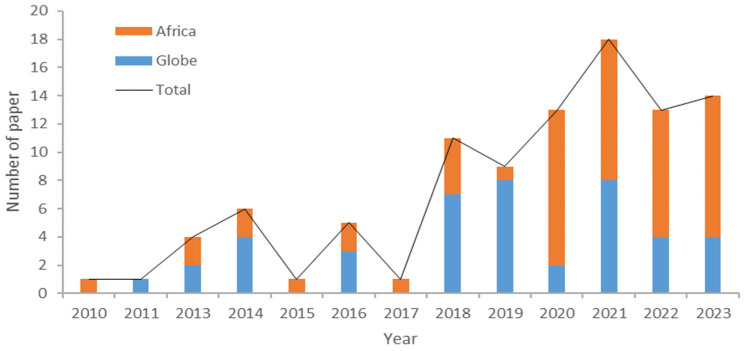
Number of publications per year from 2010 to 2023.

The initial systematic search using the keywords “weather extremes”, “maize”, and “Africa” resulted in 489 papers. After applying the selection criteria (Figure 1), the search was narrowed down to 96 papers, 42 of which were global studies containing implicit information for Africa, while 54 addressed African countries exclusively. The number of publications dealing with the impacts of climate change on agriculture, its vulnerability, and the best adaptation strategies has more than tripled between 2010 and 2023. This expanding trend has continued in subsequent years, as shown in Figure 2. This growing interest in the impact of climate change on agriculture is, in part, related to the importance of agriculture in the economies of African countries. Conversely, the heterogeneity and unpredictability of global climate change require continuous improvement in predicting and adapting to extreme weather events.

### 3.2. Article Classification

The papers were classified based on four standards: focused region, published year, addressed extreme types, and research approach. Of the 96 articles, 42 (43.8%) were from global studies, while 54 (56.2%) were from specific African countries or the whole of Africa. Due to Africa’s minor role as a maize producer in the global market, discussions on African maize were mostly implicit or limited in the majority of the studies. Consistent with the findings of Pasgarrd et al. [27], over 80% of the publications, particularly the global studies, did not include a single African author, with authorship dominated by researchers outside Africa. These imbalances in scientific production can reduce adaptive capacity in Africa as researchers at Global North institutions may shape research questions and outputs for a northern audience rather than provide actionable insights on priority issues for African farmers.

**Figure 3:**
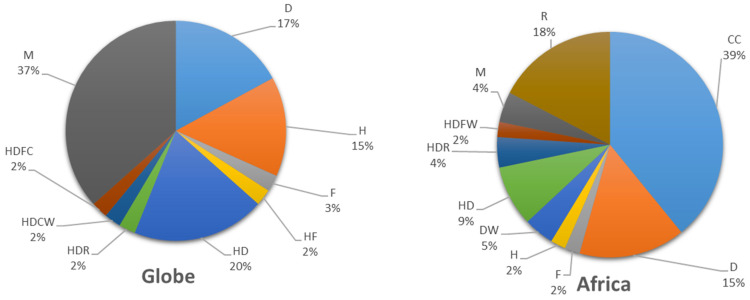
Extreme climate events addressed in the publications and their proportions (%). CC: mean climate change with extreme weather being implicitly included; D: drought; H: heatwave; F: flood; R: extreme rainfall; W: wet; C: cold; HF: heatwave and flood; HD: heatwave and drought; DW: drought and wet; HDR: heatwave, drought and flood; HDCW: heatwave, drought cold and wet; HDFC: heatwave, drought, flood and cold; HDFW: heatwave, drought, flood and wet; M: multiple events (more than four).

The first study specifying weather’s extreme impact on maize yield was conducted by Lobell DB [17]. The number of studies increased substantially after 2015. Although similar studies in Africa started in 2010, there are few studies specifying the impact of extreme weather on maize yield. Since 2020, this trend has reversed, as shown in Figure 2 with a dramatic increase in the number of papers and publications on this topic, many of which have concentrated on the two key agroclimatic extremes: drought and heat. Some publications addressed two or more extreme weather events, like HD (heat and drought), and HDCW (heat, drought, cold, and wet). Some studies, particularly the African studies that only researched the impacts of mean climate change but with a general discussion of extreme weather events, were also included. There has been an increasing number of studies since 2000 that have examined the effects of multiple extremes, particularly the compounding effects of multiple stresses, such as heat and drought. Nevertheless, such research is still deficient in Africa, where only 20% of African studies examined the impacts of multiple weather extremities, with the majority of studies in Africa exploring singular extremes independently, as shown in Figure 3.

### 3.3. Methods Used to Estimate the Effects of Weather Extremes

**Figure 4:**
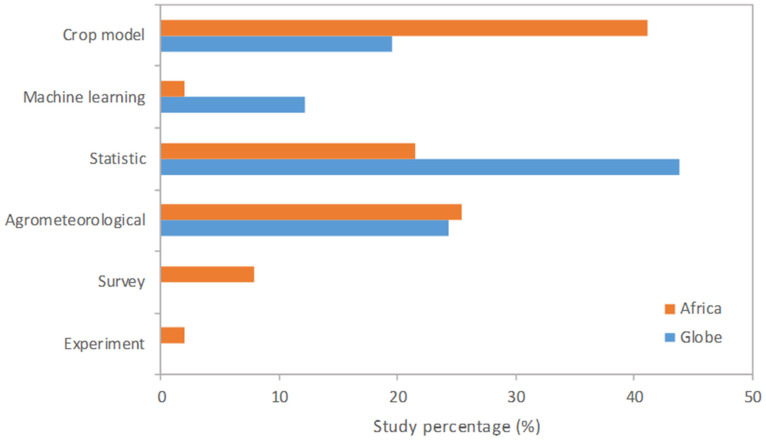
Investigation methods used in the literature.

Researchers use a variety of methods to study the impacts of weather extremes on crop production. Common research methods include field experiments, remote sensing, and statistical analyses to assess important factors, such as temperature fluctuations, precipitation patterns, and extreme events. The literature describes roughly six types of methods used to quantify the effects of extreme weather, as follows (Figure 4):

- Experimental: These studies collect on-farm data over extended time periods to directly measure crop productivity under different weather conditions and extreme events, like drought and heatwaves. Researchers quantify yield variability and decline during years with extreme weather.
- Agrometeorological: the values of the explanatory variable accounting for the extreme magnitude are plotted against observed maize yield or area damages.
- Statistical: Researchers analyze historical crop yield data alongside weather, climate, and environmental datasets using statistical models to estimate relationships and correlations. This allows for the assessment of what specific temperature, water, and climate factors influence crop yields or productions.
- Process-based modeling: crop growth models have been developed to simulate crop development under projected future climate scenarios. These models, like DSSAT and APSIM, incorporate data on genetics, soil, weather patterns, and environment to estimate future yields. They allow for the testing of adaptation strategies.
- Survey: farmer or expert information is used to study weather extremes and damage.
- Machine learning: this method is similar to statistical analysis, but researchers use various algorithms, such as artificial neural networks or random forests, to quantify the damage.

These studies of observed impacts use historical data on climate, production area, and yield, and they identify the role of extreme weather in driving changes in suitability, production, yield, food quality, or total factor productivity. Quantitative analysis is only possible in places with adequate historical data, such as long-term field observation or crop trials. The fundamental knowledge of weather stress impacts on crops originated from field experiments on maize responses to various conditions and perturbations to environments, such as TFACE and FACE experiments, which have been used to develop crop models and understand the effect mechanisms of extreme weather. Nonetheless, this type of research has dwindled in prevalence in recent years due to its high expenses, prolonged duration, and constraints, like scalability challenges. In Africa, long-term experimental infrastructure is lacking, resulting in a limited understanding of the impacts of climate change and extreme weather in low-input, tropical, or semi-tropical environments. One of the few studies with an experimental method conducted in Africa, as reported in the literature review, is Lobell et al. [17], who used data from CIMMYT’s long-term breeding trials to quantify the heat stress impacts on maize yields, where the use of “environmental mega data” is one of the challenges. In many African cases lacking reliable long-term observed data, studies rely on qualitative assessments, often drawing on farmers’ perceptions of climate impacts.

The agrometeorological method has been widely used to examine the impact of weather extremes. This method has evolved from the temporal and spatial analysis of weather data, i.e., from simply linking crop cultivation and plantation to regression between agrometeorological indices and reported crop yields, to further incorporating these indices into statistical and spatial models. The vulnerability assessment of maize production to climate change is a type of research that relies on the index approach, in which sensitivity, exposure, and adaptive capacity indices are represented by various spatial variables [28]. Other applications, at the global scale, include Arnell et al. [29], who found that based on temperature indices, the chance of a damaging hot spell for maize increased from 5 to 50% at 4 °C rise. Trend analysis of the dry spell index for maize production in South Africa found no significant trend in the past [30]. Heatwaves and extreme precipitation showed no significant trends, but drought severity increased in some provinces. Regression analysis demonstrated that the historical national maize yield in South Africa was associated with drought events, explaining 25% of maize yield variability. In contrast, heatwaves or a combination of droughts and extreme precipitation have a significant influence on maize yield variability in some states [16]. Chemura et al. [19] utilized the MaxEnt suitability model and several agrometeorological indices to predict maize suitability up to 2064. Extreme climate indices in maize suitability modeling improved the efficiency of the maize suitability models and showed more severe changes in maize suitability over Southern Africa than using season-long climatic variables. In addition, agrometeorological methods, such as are used for Partellus, have also been used to predict maize insert distribution under climate change [31].

Statistical approaches are another major method used around the world and in Africa to measure the impact of extreme weather on maize. Rich historical data have made statistical analysis possible, enabling it to be used for a wide range of temporal, spatial, and attribution analyses. Simply statistical analysis, such as time series analysis of past grain yields, has detected a decreasing trend of yield variability in cereal crops across the globe; yield decreases in some regions, such as maize in Kenya and Tanzania, have indicated increasing instability in maize cultivation [32]. Utilizing specific statistical models, such as superposed epoch analysis and global emergency events data plus national crop production data (production, yield, and harvest area), Lesk et al. [33] quantified the national cereal production losses (1964–2007) due to four weather extremes—drought, heat, flood, and cold. They found that production losses due to droughts were associated with a reduction in both harvested area and yields, whereas extreme heat mainly decreased cereal yields. The analysis also revealed ~7% greater production damage from more recent droughts. Some statistical models include economic factors, such as GDP, with panel data in Africa, but most focus on changes in mean climate variables [34] due to the rare occurrence of climate extremes in history.

A subgroup within the statistical approach is the machine learning method (ML), which has seen a substantial increase in recent years due to advancements in computational power and AI techniques. This approach can handle large datasets, typically using historical data to create ML models and then applying the models to a variety of future scenarios, such as climate, management, and socioeconomic development. A comprehensive study employing the ML method estimated that global maize losses will substantially increase due to extreme heat and drought events within a 10-year return time during the period 1980–2011, becoming the new norm at 1.5 °C global warming levels (approximately 2020s). At 2 °C warming (late 2030s), maize-growing regions will experience heat and drought stresses that have not been observed before, impacting both major and minor production areas [35]. Using gridded yield data and ML approach, Vogel et al. [36] estimated that historical climate (1961–2018), including mean conditions and extremes, explained 20–49% of the variance in yield anomalies, with 18–43% of the explained variance attributable to climate extremes. Temperature-related extremes demonstrate a stronger association with yield anomalies than precipitation-related factors. Irrigation partly mitigates the negative effects of high-temperature extremes. Chemura et al. [37] used the ML approach to investigate the climatic suitability of crops under climate change in Ghana. They found that precipitation-based factors are the most important determinants of crop suitability. The optimal suitability for maize will decrease by up to 12% under RCP2.6 and 14% under RCP8.5. However, the number of ML studies in Africa remains limited due to the constraints in skills, models, and data availability.

In both Africa and globally, the projection of future impacts of weather extremes and the proposal of adaptation strategies rely on process-based models that combine climate data with data from experimental studies to test how crops respond to different climate or management factors. Studies with crop models in Africa have increased substantially since 2015 due to some model intercomparison projects, such as AgMIP (the Agricultural Model Intercomparison and Improvement Project) and the ISI-MIP (Inter-Sectoral Impact Model Intercomparison Project). Based on these projects, the Global Gridded Crop Models (GGCMs) have been used to identify the impacts of droughts and heat waves on crop yields. Specifically, the drought signal was detected by 12 out of 13 models for maize, while 11 models detected the heat signal for maize. An intercomparison experiment utilizing 25 models was conducted in low-input maize systems across 5 sub-Saharan countries. The simulation revealed that nitrogen fertilization controlled the response to variations in CO_2_, temperature, and rainfall. Without N fertilizer input, maize exhibited less benefit from increased atmospheric CO_2_, was less affected by higher temperature and decreasing rainfall, and was more affected by increased rainfall due to the increased significance of N leaching. The intercomparison model also revealed that the simulation of daily soil N supply and N leaching is crucial for accurately simulating the impacts of climate change on low-input systems [38].

Crop models have been frequently employed to investigate the yield variability changes across sub-Saharan Africa, finding increased variability and mean maize yield in most maize-growing areas [39]. These models can also be linked to agrometeorological indices to comprehend the climatic factors contributing to yield loss at a regional scale. Kamali et al. [40], using EPIC and drought indices, found that Southern African countries and some regions of the Sahelian strip are highly vulnerable to drought due to increased water stress, while Central African countries are more susceptible to temperature stresses. The crop model approach proves advantageous for examining GxExM interactions and, consequently, formulating suitable adaptation strategies. For instance, using the DSSAT crop model, Cecil et al. [41] observed that fertilizers have significant impacts on maize yield in Zambia under favorable weather conditions, whereas adjustments in planting dates exert a more substantial influence during adverse weather conditions.

However, the fact that most crop models systematically underestimate yield responses to heat and drought not only misrepresents exposure but also suggests that capacity needs to be improved to adequately respond to extreme weather events [42]. Using crop models with different improvements, such as conventional CropSyst and modified CropSyst, is an effective method to quantify the effects of extremes. The modified crop model explicitly considers the impact of extreme heat and drought. The difference between the simulations suggests heat and drought can cause an 8–21% additional yield decline in maize in South Africa [43,44]. Nevertheless, extreme weather affects crops through multiple mechanisms, including climatic and crop coupling, crop biophysical responses, and interactions with other factors, like management (e.g., waterlogging, late harvesting, and fertilizer leaching), that cannot be fully captured by crop models. This suggests that further field experiments are needed across different cropping systems to enhance the accuracy of predictions, particularly for the low-input systems in Africa.

In summary, past studies have employed a variety of approaches to assess the impacts of climate change on agricultural yields in Africa, ranging from simple comparisons of future impacts to historical drought-induced yield losses [45] to more sophisticated crop simulation modeling [46], statistical time series analysis [47], and cross-sectional analysis [48]. For Africa, simulation and statistical approaches are the two major methods employed. To date, simulation studies have been limited by a lack of reliable data on soil properties and management practices, providing only ‘best-guess’ estimates with little to no information on uncertainties stemming from choices in model structure, parameter values, and scaling techniques [49]. Statistical analyses have been limited by the poor quantity and quality of historical agricultural data relative to other regions, resulting in model estimates with wide confidence intervals [47].

## 4. The Current Understanding of Weather Extreme Risks and Their Impacts on African Maize

### 4.1. Changing Trends in Agrometeorological Indices

Temperatures are expected to increase in West, East, and Southern Africa, with multi-model climate projections indicating a warming of 1–4 °C between 2081 and 2100 relative to the period between 1986 and 2005, depending on the representative concentration pathway (RCP) considered [23]. This warming trend is projected to be faster in Africa than the global average. Changes in annual rainfall across SSA are uncertain but are expected to increase in West and East Africa (0% to +12% depending on RCP) and to decrease in Southern Africa (−5% to −10% depending on RCP). The impact of climate change on maize productivity across SSA is also uncertain, but significant losses are expected, especially in Southern Africa [50,51] and West Africa [52].

Several studies, including the IPCC reports, have reviewed changes in mean climate and extreme events in Africa. Estimated warming rates vary depending on the time period and data used. For example, an FAO study indicates that from 1990 to 2018, temperatures in SSA increased by 1.6 °C–2.0 °C, while precipitation declined by 4% [53]. With increased greenhouse gas emissions, the mean temperature is projected to rise by 1.0–3.0 °C by 2060 across the entire continent, as are temperature extremes over most of the continent. Increased mean annual rainfall is projected over the eastern Sahel, eastern East Africa, and Central Africa. Conversely, reduced mean annual rainfall and increased drought are projected over southwestern Southern Africa and coastal North Africa, driven in part by an increasing atmospheric evaporative demand due to higher temperatures. The frequency and intensity of heavy precipitation are projected to increase across most of Africa, except for in northern and southwestern Africa.

Drought has been the most prevalent disaster impacting regional food insecurity, particularly in Southern and Eastern Africa. For example, Eastern Africa has experienced eight droughts from 2001–2016, with the drought frequency doubling from once every 6 years to once every 3 years since 2005 [54]. In addition to drought, African farmers perceive a wide variety of climate threats to crop production, including precipitation variability, delayed onset of rains, reduced early-season rainfall, and excessive heat [55]. These perceived changes are seen as a major driver of yield losses by farmers [56]. Over half of surveyed farmers in West Africa attribute increases in crop pests and diseases to climate change, as the range and seasonality of many pests and diseases change under warming [57]. Pests and diseases contribute to yield losses of 10–35% for wheat, rice, maize, potato, and soybean in sub-Saharan Africa [58]. The recent locust outbreaks in 2019 in East Africa have been linked to climate conditions, partly driven by ocean warming [59].

A defining feature of extreme weather patterns in Africa is the teleconnection of extreme impacts across regions, driven by specific atmospheric circulations, like the El Niño–Southern Oscillation (ENSO). The ENSO is a major driver of climate variability with direct consequences for agricultural production in Africa, causing frequent droughts during past decades in Eastern and Southern Africa [60]. Maize yield, specifically, tends to decrease during the positive phase of ENSO (El Niño) in much of Southern Africa, while yield tends to increase during the positive phase in Eastern Africa [61,62].

**Figure 5:**
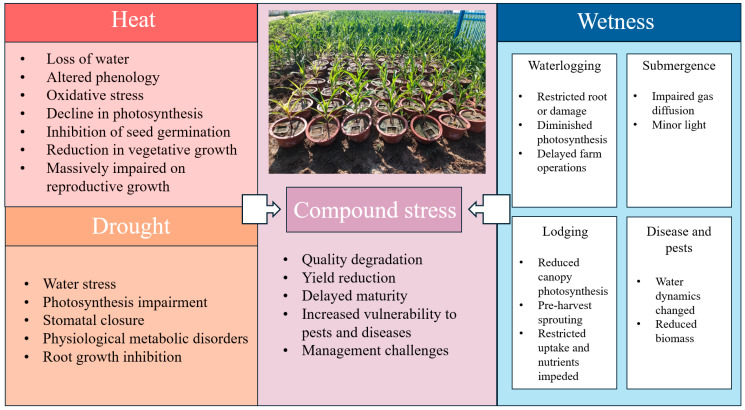
Maize yield loss mechanisms are due to three types of extremes and compound extremes (i.e., heat and drought, heat and wetness).

The review identified only 42 papers from Africa, with even fewer studies specifically isolating the impacts of weather extremes on African crop production. A limited number of studies, primarily in South Africa, have recently emerged to investigate the effects of heat, drought stress, or compound extreme events on maize production, employing methods, such as agrometeorological indices [15,16], crop suitability model [19], and crop models [44]. The literature extensively explores the physiological response mechanisms of maize to a range of adverse and extreme agroclimatic events, including heat, cold, drought, heavy rain, and flooding (e.g., Lesk et al., 2022 [63]; Pokhrel, 2021 [64]; Waqas et al., 2021 [65]; Rezaei et al., 2015 [66]; Sanchez et al., 2014 [67]; Sha et al., 2017 [68]). The following section summarizes maize’s response mechanisms to three types of extremes (Figure 5) considered in these reviews, providing a rough estimation of the observed and projected impacts on Africa based on both African and global studies containing information about Africa.

### 4.2. Drought

Drought is the most concerning climate disaster in Africa because of its large rainfall variability and the rain-fed and low-input agriculture conducted by smallholder farmers.

Affecting Mechanisms: According to the definition in the IPCC Report, drought is a period of abnormally dry weather long enough to cause a severe hydrological imbalance. Drought is a relative term and any discussion in precipitation deficit must refer to the precipitation-related activity that is under discussion. The scientific literature commonly distinguishes meteorological drought, which refers to a deficit of precipitation, soil moisture drought, which refers to a deficit of soil moisture, and hydrological drought, which refers to negative anomalies in streamflow, lake, and/or groundwater levels [69]. Here, we only address soil moisture drought, or agricultural drought.

Drought is one of the types of natural disasters with the widest effect [70], and it damages maize by affecting its growth, reproduction, and physiology. Field experiments showed that drought with approximately 40% water loss reduced wheat yield by 20.6% [71]. Drought stress can reduce overall plant size, ear size, and kernel number. While drought starts to affect maize growth during the vegetative growth stage, it mainly poses detrimental effects on the final yield during pollination and fertilization. For example, if drought stress continues for two weeks before pollination and the plant wilts, yield can be reduced by up to 3–4% per day [72]. During the silk and pollen development stage, yield losses can be up to 8% per day; after silking occurs, yield losses can reach up to 6% per day. One mechanism for the high sensitivity to drought during pollination is that drought can disrupt the synchrony between pollen availability and silk emergence. Drought also dries out exposed silk, making it unable to accommodate captured pollen grain. Drought causes considerable delays in maize female organ development. During the reproductive stage, water shortages can result in the inhibition of photosynthesis, reducing the nutrients supplied to generate organs. In addition, reduced soil moisture can further increase the likelihood of heat stress [73].

Observed changes and impacts: From 1951 to 2011, a clear drying tendency dominated global grain production areas, including increased drought duration, the number of impacted areas, and the severity of hazards. Drought disasters account for nearly 20% of all disaster occurrences in all African regions, affecting a large percentage of people, especially in rural communities. An increase in agricultural drought is likely to occur in almost all the regions of SSA, but much more so in East Africa, Central Africa, and some parts of Southern Africa due to slow progress in drought-risk management [7].

Maize in sub-Saharan Africa, particularly in Central, Eastern, and Southern Africa, exhibits high susceptibility to complex drought patterns, including different drought durations and timing throughout the entire farming season [74]. Studies show that drought, especially in relation to EI Niño events, has been a major contributor to maize yield losses and food insecurity in parts of Africa. It is estimated that South Africa experienced 10–25% yield losses during the EI Niño years [15].

Projected changes and impacts: Drought can coexist in different forms (e.g., meteorological, hydrological, and agricultural) as concurrent droughts. Such concurrent droughts can have far-reaching implications for crop yields and global food security. By using standardized indices of precipitation, runoff, and soil moisture incorporated through the multivariate standardized drought index (MSDI) with copula function, Muthuvel et al. [69] quantified the concurrent droughts and their effects on global maize yields. South Africa has been identified as one of the most vulnerable regions for droughts in the 21st century. The conditional concurrent drought probabilities will increase in South and East Africa, suggesting that concurrent drought will affect their maize yield tremendously in the far future.

Projections indicate a substantial increase in drought risk for key maize-growing regions in Africa under climate change. Countries in Africa, except for Eastern Africa, tend to become dryer, exhibiting higher susceptibility to extreme droughts and suffering more losses than developed countries and regions [75]. According to IPCC AR6, maize yield is predicted to decline up to 17% for 1.5 °C warming and 33% for 4 °C warming in Africa [23]. Drought is also projected to affect maize production in Ethiopia, particularly under a high GHG emission pathway [76]. Research coupled climate and crop models to simulate maize growth under drought. The results indicated that parts of Kenya could see declines of over 25% in suitable maize cropping areas by 2050 due to increased drought frequency (MPI). Drought and its nonlinear interaction with heat stress [32] occur earlier in the growing season. In South Africa, drought can decrease maize yields either in the year of the drought or in the subsequent year, depending on the exact timing of the low-rainfall events in the season and soil moisture storage [77].

Gaps: Although maize traits related to maize yield under water deficit have been well established, consistent progress toward greater yields under drought has been difficult to obtain [78]. Many of the gaps are associated with the difference in the timing of water stress relative to crop development and with genotype-by-environment interactions. With drought-tolerant cultivars becoming a solution to cope with drought in Africa [79], knowing the long-term frequency with which drought-stress seasonal patterns occur will help breeding programs to decide target traits for selection and seed markets in targeting locations for new lines dissemination. However, a detailed analysis of drought stress seasonal patterns occurring both under current and future conditions, and any associated changes in the frequency of the main stress types, as well as the link with atmospheric circulation patterns, such as ENSO, is still lacking for SSA.

### 4.3. Heat Stress

Affecting Mechanisms: Generally, for crops, heat stress is often defined as the rise in temperature beyond a threshold level for a sufficient period of time to cause irreversible damage to plant growth and development. Numerous researchers utilize the term “heat waves”, describing them as extended periods of extreme heat; however, there is no uniform definition concerning the temperature threshold, temperature metric, and duration applied to delineate heat waves. Certain studies have employed thresholds based on mean temperature, apparent temperature, or combinations of apparent and minimum temperature thresholds [80]. Consequently, the inconsistent application of heat wave definitions resulted in varying time frames being labeled as heat waves, impeding the comparability and synthesis of findings among studies. In the majority of the reviewed literature, heat stress was characterized by an elevation in mean daily temperature surpassing a specific threshold, like 32 °C, integrating shifts in mean temperature and the occurrence of heat waves.

Temperature’s effects on maize are multi-faceted. On the one hand, increases in temperatures cause greater water loss from the soil due to increased evapotranspiration, thus reducing plant water supply. On the other hand, increases in temperature increase plant transpiration and photosynthesis, which concurrently increase plant water demand, potentially up to the point of plant desiccation [81]. High temperatures also have the effect of reducing maize pollen fertility, hastening grain filling, and increasing wasteful respiration, resulting in a yield decrease of about 7% for every degree of warming [67]. In addition, higher temperatures alter the required growing degree days, reducing grain filling duration.

The optimal and lethal temperatures for maize growth have been determined based on field observation or controlled experiments. For example, the optimum daytime temperature to grow maize ranges between 28 °C and 32 °C, with the top thresholds of 38.9 °C for shoot development, 39.2 °C for tassel initiation, 37.3 °C for anthesis, and 36 °C for grain filling [67]. Temperatures above the threshold for various metabolic, biochemical, and physiological processes result in an imbalance for these activities and activate the innate plant defense system. Temperature extremes alter the photosynthetic process, damage the biological membranes, affect nutrient uptake, and limit the functioning of various enzymes in maize. Stunted growth and low photosynthetic rates cause impairment in overall maize performance. High seasonal temperature anomalies accelerate maize phenological phase transitions, reducing yields [82,83].

Maize is extremely sensitive to heat waves during the flowering period. Heat stress during the reproductive phase causes desiccation of silks, pollens’ sterility, and poor seed setting, resulting in drastic yield reduction [67]. Productivity loss at the reproductive phase due to heat stress is also linked with a decrease in the number of grains and their weight [80]. The reduction in grain amount and weight is attributed to reduced photosynthesis, increased respiration, as well as direct negative impacts on reproductive processes due to the high temperature. Specifically, heat stress from the ninth leaf stage to the tasseling stage can slow tassel growth, destroy other structures, and reduce pollen viability. During the grain filling period, high temperatures shorten kernel filling and decrease yield. Since maize is left in the field to dry up before harvesting, it is usually not sensitive to heat stress in the final period of the cropping system.

Observed changes and Impacts: Due to climate warming, exposure to temperatures above maize’s optimal temperature has increased around the globe, particularly after 1980 [84]. For example, from 1981 to 2011, areas with exposure of maize to temperatures above 37 °C were widespread and severe, expanding from the subtropics to mid-latitudes to encompass major global maize-producing areas. In Africa, temperature increases have been detected across the whole continent, and many regions have warmed more rapidly than the global average. A sign of increased annual heatwave frequency has emerged from the background natural climate variability over the whole continent, but a significant positive trend has not been detected in most of the crop areas in Africa [23].

Because heat waves are rare in historical weather, most studies focus on increasing mean temperature. Increasing temperatures are already harmful to crop production and are slowing productivity growth in Africa. Changes in mean climate have reduced total agricultural productivity growth in Africa by 34% since 1961, more than in any other region. Maize yields have decreased 5.8% on average in sub-Saharan Africa due to climate change in the period 1974–2008. Overall, climate change has decreased total food calories across all crops in sub-Saharan Africa by 1.4% on average compared to a no-climate-change counterfactual since 1970, with up to 10% reductions in Ghana and Zimbabwe. Using a dataset of more than 20,000 historical maize trials in Africa, combined with daily weather data, a study shows a nonlinear relationship between warming and yields. Each degree day spent above 30 °C reduced the grain yield by 1% under optimal rain-fed conditions and by 1.7% under drought conditions [17].

Projected changes and impacts: The increase in heat stress is projected to increase substantially in the future [85]. By the 2050s, the total area exposed could increase by up to 195% to 308.2 million ha, of which areas exposed for over 60 days may increase nearly seven-fold. The average length of exposure may increase by 69% to 27 days, and areas optimally suited to planting maize may see the fastest increase by up to 772% [86]. This extreme heat stress at anthesis by the 2080s under high emission scenarios (RCP8.5), taking into account CO_2_ fertilization effects, could double global losses of maize yield [87].

Most African countries that were expected earlier in this century to experience unprecedented high temperatures in their recent history are generally wealthy and high-latitude countries. As lower latitudes have lower internal climate variability, the low-latitude African countries are projected to be exposed to large increases in the frequency of daily temperature extremes (hotter than 99.9% of their historical records) earlier in the 21st century compared to generally wealthier, higher-latitudes nations. Although higher warming rates are projected over high latitudes during the first half of this century, societies and environments in low-latitude, low-income countries are projected to be exposed to unprecedented climates earlier than those in high-latitude, developed countries. For example, beyond 2050, in Central Africa and coastal West Africa, 10 months of every year will be hotter than any month in the period 1950–2000 under a high emission scenario (RCP8.5).

As the largest maize producer in the continent, Southern Africa’s maize regions are at risk of experiencing record-breaking hot, cold, dry, or wet events under current climatic conditions. The annual chance of unprecedented high temperatures is approximately 4%, increasing to 62% during very strong El Niño years [15]. The results are limited in enabling an understanding of what the future of agriculture will be, as illustrated by the variation in expected impacts as a function of the study methodology.

### 4.4. Heavy Rain/Flooding/Waterlogging

Affecting mechanism: Compared to drought impact, the impact of excessive rainfall and wetness on crop yield remains unresolved. Less attention has been paid to excessive rainfall or waterlogging, despite available field and experimental evidence indicating that excessive water reduces crop production as often as deficient water [88]. Estimates of yield loss due to extreme rainfall need to assess the magnitude of extreme rainfall and timing of crop exposure, both of which are highly heterogeneous over space and time. Spatially, previous studies indicate that yield statistics and climate variables aggregated at administration zones have probably smoothed out the highly localized extreme rainfall events, which could have resulted in a non-significant and weaker impact of extreme rainfall than heat and drought reported based on national statistics [89]. Temporarily, the exposure of crops to extreme rainfall can be dismissed if climate data are used with a coarse temporal resolution to explore the climate–yield relationships. The lack of clear mechanistic evidence for extreme rainfall impacts has also led crop modelers to dismiss their potential effects in projecting the impacts of climate change, despite the expected increase in occurrences of extreme rainfall [90].

Mechanistically, the precipitation responses of crop yield are more complicated and heterogeneous, as they reflect the integrated outcome of multiple plant physiological, biochemical, and soil hydrological processes. Extreme amounts of precipitation and water excess in the soil can reduce crop yield through several biophysical processes: waterlogging, submergence, lodging, and vulnerability to pests and diseases (Figure 5). Yield loss related to waterlogging is associated with delayed farm operations [91], poor crop establishment, root damage reducing water and nutrient uptake [92], impaired photosynthesis [93], and exacerbated nitrogen loss by leaching and denitrification [94]. Submergence (i.e., roots and part or the full shoot are underwater) subjects crops to limited gas diffusion and reduced light in addition to the waterlogging stresses. Excessive rainfall, accompanied by strong winds, causes plant logging, resulting in reduced canopy photosynthesis due to altered canopy architecture [95], reduced nutrient translocation due to bent or broken stems, pre-harvest sprouting, and harvest loss, reducing the overall yield. Different types of crop pests and diseases benefit from excess water to infect and develop, and cause, as a consequence, yield losses due to reduced plant biomass, impaired photosynthesis, and altered water dynamics in the soil–plant–atmosphere system [96].

Observed changes and impacts: The Food and Agriculture Organization (FAO) of the United Nations reported that floods were the second gravest agricultural disaster, next to droughts, as they were responsible for USD 21 billion in crop and livestock loss from 2008 to 2018 in the developing world [97]. A correlation analysis between the global river and inundation output and global historical yield revealed that for return periods over ten years, global average yield losses were estimated to be 1% for maize, which is less than soy (4%), rice (3%), and wheat (2%). However, East African maize production suffered from increased drought and wet events from 1981 to 2017, with climate variability explaining over 50% of maize yield variation [20].

Crop models have been used to investigate the precipitation on maize yield in Ethiopia. They showed that more precipitation is likely to harm the crop yield in Ethiopia [98]. Flood impacts on maize yield are underestimated by crop models. On-farm experiments in the West African Sahel found that between three days and six days of flooding reduced maize yield by at least 35% when they occurred during the tasseling stage. Only 4–6 days of flooding reduced maize yield by 21% at the six-leaf stage [99]. However, the large, detrimental impacts of flood or wet extremes are largely underestimated by most process-based models. In Ghana, under wetter-than-normal rainfall conditions, 25% of the variation in maize yield could be attributable to non-climatic conditions or practices, while climatic parameters account for 40.8% of maize yield under drier-than-normal rainfall conditions [100].

Projected changes and impacts: The IPCC reports revealed that by the end of this century, in a 4 °C warming scenario, sub-Saharan African countries will face many challenges to their food systems [23]. Projections show extreme precipitation intensification will increase over many parts of sub-Saharan Africa. For example, CMIP6 projects that the maximum of consecutive 1-day precipitation amount will increase by 6% with global warming of 1.5 °C and will further intensify to 12% and 22% for 2 °C and 3 °C of global warming, respectively. Similarly, the maximum number of consecutive 5-day precipitation will increase over sub-Saharan Africa by 6%, 10%, and 20% for 1.5 °C, 2.0 °C, and 3.0 °C global warming, respectively. Increased extreme precipitation triggers heavy rains and floods, which will damage crops and infrastructure and cause harvest delays in Africa [101].

The impact of excessive rainfall on crop yield can be complicated by other confounding factors. Focusing on extreme conditions (e.g., extreme wet or dry years) when crop yield significantly departs from its trend can improve the detection and attribution of extreme climate impacts. However, doing so also makes the quantification inherently dependent on the definitions and occurrences of such events. In addition, the occurrence of excessive rainfall and its impact is more localized than drought, as it involves local factors, such as soil properties, topography, and water table depth. Previous studies revealed that excessive rainfall, which has been largely under-studied previously, can adversely affect maize yield as much as extreme drought, especially at a regional scale. It is not only the major cause of crop damage for maize but also has broad impacts on other staple crops and will play a more important role in the future given the projected significant increase in extreme rainfall. As such, it is important to improve our understanding and modeling of excessive rainfall impact, which is not only pivotal in providing accurate predictions of climate change impacts on agriculture but also developing effective management and adaption measures (e.g., drainage or levee systems, cultivars) to mitigate crop yield loss.

### 4.5. Compound Stresses

Types of compound stresses: There are three types of compound extremes: compounds of a specific extreme of different types, such as meteorological, hydrological, and agricultural droughts [69]; concurrence of different types of extremes, such as hot–dry or cold–wet [102]; and simultaneous crop failures in multiple global breadbaskets for specific crops [103]. The compound extreme of a specific extreme is described in the above section. This section mainly addresses the latter two types of compound extremes.

Observed changes and impacts: Extreme heat, drought, and moisture excess are increasingly co-occurring within a single growing season, impacting crop yields. Compound heat and moisture extremes have become more intense and frequent in many cropping regions since the mid-twentieth century [104]. Compared with the mid-twentieth century, compound hot–dry extremes during maize-growing seasons have increased around the globe [63]. In particular, since approximately the 1950s, the global frequency of such events has roughly doubled [105]. Over the same period, the mean annual cropland area exposed to such events has increased by 1–2% per decade [104] relatively evenly across major breadbaskets. Positive trends in hot–dry extents have accelerated since approximately the 1980s and are largely attributable to warming temperatures more so than changes in precipitation [105]. Compound hot–wet events have also increased in frequency. Compared with the mid-twentieth century, the probability of compound flood and heat events has increased globally by roughly a factor of 2–3 times [106]. Since 1979, extremely humid heat events have also doubled in frequency and intensified over most cropping regions [107,108].

For crops, compound dry–hot events could cause larger impacts on maize yields than individual droughts or extreme heat could. For example, at the global scale, changing from extreme drought to compound dry–hot weather increases the probability of maize yield reduction from 0.07 to 0.31; while changing from extreme hot to compound dry–hot weather increases the probability of maize yield reduction from 0.04 to 0.31 [109]. From 1970–2013, global maize yield dropped more during hotter growing seasons in places where decreased precipitation and evapotranspiration more strongly accompany higher temperatures, indicating the potential influence of compound heat–drought phenomena on crops [110]. A global statistical analysis showed that the relationship between climate and maize yield in the top ten maize-producing countries has changed from 1961–1988 to 1989–2016. The risk of crop yield reduction generally increases with enhanced dependence between dry conditions and crop yields (or hot conditions and crop yield). The risk of maize yield reduction increases for the most of these countries under compound dry–hot conditions due to concurrent changes in climate-yield relationship [111,112]. Similarly, from 1980 to 2009, co-occurring global extremely hot and dry events have consistently negative effects on crop yields, while extremely cold and wet conditions, though they also reduced crop yields, impacted yield to a lesser extent, being more uncertain and inconsistent [102].

In Africa, since 2000, several specific cases of compound heat and moisture extremes have been linked to severe yield losses. For example, the 2015–2016 security crisis in Southern Africa further deteriorated due to a flash drought that was intensified by heat waves [113]. In Ethiopia, low cereal yield from 1961 to 2014 (>1 standard deviation below the mean) was associated with a combination of heat (83rd percentile temperature) and drought (~20th percentile precipitation and runoff) [114]. In 2022, a multi-year drought combined with record-high temperatures led to crop failures, exacerbating food crises in many districts in East Africa [63].

However, despite some existing evidence for the risks of warm droughts, shown by its impact on crop photosynthesis since the 1980s, the effects of the rise in compound extremes on global crop yields overall remains underexamined in some areas. Climate trends explain increases in year-to-year yield variability since the 1980s, including maize in East Africa. Increasing compounding among various climate influences on crop yield has probably contributed to the changes, but how and to what extent are not well quantified.

Under normal climatic circumstances, the global food system can compensate for local crop losses through storage and trade, even if a region suffers from a compound climate extreme. However, it is doubtful whether the global food system is resilient to more extreme climatic conditions, especially when export restrictions and diminished grain stocks undermine liquidity in agricultural commodity markets, resulting in higher price volatility [115]. This is particularly the case for Africa. For instance, the food price crisis in 2008 showed that climatic shocks to agricultural production contributed to food price spikes and famine in Africa, with the potential to trigger other systemic risks, including political unrest and migration [116]. Climatic teleconnections between global phenomena, such as the El Niño–Southern Oscillation, and regional climate extremes, such as Indian heatwaves or flood risks around the globe, could lead to simultaneous crop failure in different regions [117], therefore posing a risk to the global food system and amplifying threats to global food security. Indeed, studying past data from 1961 to 2016 revealed that on a worldwide scale, there has been a substantial increase in the probability of multiple global breadbasket failures for all crops except rice. Looking at the extremes, the annual probability of all breadbaskets experiencing climate risks simultaneously increased from 0.8% to 1.1% for maize [103]. Furthermore, this risk of multiple breadbasket failures for maize will increase from 6% to 40% at 1.5 °C and to 54% at 2 °C warming [115], and further under 4 °C warming [118]. While a few global studies have started to look at the increasing possibility of a climatic extreme hitting more than one breadbasket for both historical and future climate conditions, no studies so far have examined their consequences and adaptation requirements for Africa, even though Africa is the weakest point in the link that may suffer the most in the supply chain [119,120].

Projected changes and impacts: For the future, compound extremes are projected to continue intensifying under a wide range of plausible future emissions scenarios [121]. Under a warming of 2.25 °C, the chance of concurrent heat and drought leading to simultaneous maize failures in three or more breadbasket regions approximately doubles, while inter-annual wet–dry oscillations are 20% more likely across much of the subtropics [122]. This concurrent damage across multiple breadbaskets may result in unrest and food insecurity in parts of Africa that depend on imports, suggesting that a global assessment of crop failures and their predictability is needed beyond the regional or national level [123]. However, understanding the implications of these changes for crop yields is in its infancy and subject to considerable uncertainties. Nevertheless, a consensus is emerging over certain dimensions of yield risk and opportunity from compound extremes. These risks and opportunities will largely be determined by responses to the three modes of compounding interactions: crop–physiological, heat–moisture, and crop–atmosphere interactions [63].

## 5. Adaptation Options and Assessment of Their Effectiveness

The necessity for adaptation to climate change and climate-related hazards has attracted the attention of researchers in SSA. Magesa et al. [124] conducted a systematic review to evaluate the smallholder farmers’ choices and adoption measures to reduce the effects of climate change. This study analyzed adaptation measures based on 66 associated studies, consisting of 4 categories: (i) crop varieties and management; (ii) water and soil management; (iii) financial schemes, migration, and culture; and (iv) agriculture and weather services. The dominant strategies identified are crop diversification, planting drought-tolerant varieties [79], changing planting dates, and planting early maturing crops. These adaptation strategies are a welcoming development that may be beneficial for responding to the impact of climate change. However, they may not be effective during times of more extreme climate events in the coming decades. More transformative changes should be promoted, including building more infrastructure for irrigation, promoting crop insurance, using improved varieties, and increasing opportunities for livelihood diversification.

A systematic assessment of different adaptation strategies across the whole of SSA is lacking. Most studies address adaptation in specific countries or only at a few sites. In particular, there is a need for policies and programs to appraise adaptation options in SSA, and such policies should use inclusive rights-based approaches to help scale adaptations while minimizing maladaptation [125]. At the global scale, a gridded regression analysis found that anthropogenic factors (i.e., adaptive capacity measures, such as the human development index, irrigation infrastructure, and fertilizer use) explained 40–60% of yield loss risk variation across the period of 1981–2009, whereas the factors provided noticeably lower (5–20%) explanatory power during dry and hot shock years, suggesting crop yield loss risk was modulated by anthropogenic factors. These factors explained a high proportion (~80%) of the yield loss risk variation, suggesting that the larger role of adaptation is to increase yield resilience in Africa [126]. Some studies have tried to evaluate the adaptation options in specific sites or countries. For example, Siatwiinda et al. [127] evaluated the roles of adaptation strategies with crop models at the national level. They found that although expected temperature and extremes led to a declining maize yield in Africa, the existing gaps between water-limited and nutrient-limited maize yield in Zambia are substantially larger than the expected yield decline due to climate change. In Southern Africa, improved crops and soil fertility will be important to boost maize yield in the near future. However, towards the end of the 21st century, none of the current farm management options can avoid large yield losses due to climate change [128].

Africa may need adaptations tailored to its unique practices, such as small-holder farms, low-input farms, rain-fed farms, and specific environments. For example, agricultural and livelihood diversification are proposed as effective strategies that can be used by African households to cope with climate change and increased extreme weather events, enabling them to spread risks and adjust to shifting climate conditions [129]. This includes adjusting cropping choices, planting times, size, type, and planted area locations [130]. In southern Africa, changes in planting dates provide farmers with greater yield stability in uncertain climate conditions [131]. Changing sowing time with more drought-tolerant maize cultivars that have a longer post-anthesis phase will likely reduce the yield damage of drought under climate change [132]. In Ghana, farmers are changing planting schedules and using early-maturing varieties to cope with the late onset and early cessation of the rainy season [133]. In developing adaptation strategies, local knowledge plays a critical role in enabling livelihood diversification among rural farmers in Ghana, especially under conditions of high levels of poverty [134].

Various options are considered potentially effective in reducing climate change risks, including plant breeding, crop diversification, and crop calendar shifting. However, given the uncertainties that exist around climate projection, the need to carefully consider how short-term or incremental adaptations may facilitate or hinder larger-scale, longer-term adaptations in the future has been underscored in Africa [52]. Only a few global studies have attempted to answer this question. As an illustration, without accounting for the CO_2_ fertilization effect, adaptation using genetic cultivar change can offset the yield damage for warming below 2 °C in Africa [135]. In contrast, the effectiveness of the adaptation for increased extreme weather events is unknown. Furthermore, no studies to date have considered the increasing challenges for adaptation development in a warming world. For instance, in plant breeding, increased evidence has indicated that the past speed of developing new crop varieties was slower than the warming trend [136], undermining the capacity of adaptation.

## 6. Research Gaps and Future Improvements

### 6.1. Research Gaps

There is growing evidence from the literature that extreme weather events resulting from climate change have had and will have direct impacts on African maize production. However, the exact extreme type, magnitude, and contributions to regional maize production are largely unclear in many African countries due to research gaps, hindering the generation and implementation of appropriate adaptation strategies. We identify three gaps based on the comparison between studies from around the globe and in Africa.

- Data gaps—the lack of reliable and long-term experimental and empirical data, which is essential to quantifying the risks and facilitating the improvement of forecast models. For example, extreme weather events in Africa often go unreported when compared to those in North America and Europe. Primarily, extreme heat, rainfall, and drought, the ingredients of most extreme weather events, are monitored by weather or agricultural stations. Africa has the lowest density of operational stations of any continent. A lack of observations in Africa has limited the tracking of extreme weather events and the understanding of how those events affect crop systems. In addition, the limitation in the basic information (where crops are grown and how many of them are there) adds considerable difficulty to the quantification and evaluation of impacts and adaptation options. Furthermore, process-based crop models have been increasingly employed to assess both the sites and the large-scale impacts of weather extremes on crop production. However, one of the gaps in current studies on the application of crop models is that, in most cases, the crop model has to be calibrated against historical observations, which are usually lacking in SSA [137]. An increasing number of studies are using ensemble simulations, including multiple models and a range of inputs and parameters, to account for the uncertainty, which in turn increases the difficulty of model applications by African stockholders.
- Technology gaps—technology regarding climate change adaptation consists of multiple aspects, including risk assessment methods, analysis skills and infrastructure, adaptation technology, development capacity, and new tools and approaches. Many of them are behind developed countries or are simply lacking. For example, the number of studies using gridded modeling approaches (either empirical or crop model-based) to identify the contributions of climate change and extreme weather events to crop production is substantially smaller in Africa than in other continents, such as Europe, North America, and Asia, likely due to the lack of high-performance computation capacity or models. The evaluation of adaptation options in numerous African studies predominantly emphasizes particular agronomic practices at a field level, lacking in-depth assessments of holistic adaptation strategies addressing cropping systems or the supply chain. Although agriculture–forestry, ecosystem agriculture, and various rotations have been proposed as promising adaptation strategies in Africa, assessments for specific cropping systems are still rare in Africa. There are many reasons for these gaps, including limited funding for Africa and the lower development capacity of new technology, such as cloud computation forecasting. IPCC AR6 points out that from 1990 to 2019, research on Africa received just 3.8% of climate-related research funding globally, while 78% of this funding for Africa went to EU and North American institutions and only 14.5% to African institutions. These gaps call for technological transfer from developed nations to African countries [138].
- Knowledge gaps—Both data and research gaps contributed to the knowledge gaps regarding the climate crop nexus, particularly the interactions between maize production and extreme weather events. These knowledge gaps may lead to suboptimal incremental adaptation choices, such as cultivar change or planting date adjustment. As seen in the review, there were no studies in Africa contributing to the understanding of the mechanism and effects of drought/heat/rainfall on crops due to the lack of field or long-term experiments. Most of the knowledge regarding the quantitative effects of extreme weather events on African maize come from global studies. Since Africa is not a key maize producer in the world and its unique cultivation environments are characterized as rain-fed and low-input, the estimated production response is minor and negligible, although these changes can have great impacts on farmers’ livelihood and regional food security. In addition, knowledge to facilitate adaptation development and investment is still lacking in Africa, including detailed information about how local weather is changing and what crop traits will adapt better, the dominant extreme weather events and their spatial heterogeneity, and the interactions between extreme weather events and management regimes, such as the low-input practices and low soil nutrient conditions. Furthermore, new understandings related to specific extreme patterns, such as compounding disasters and their repercussions, the teleconnection of disasters among breadbasket, and the link to African food insecurity, are scarce in Africa. In contrast, local knowledge may play an important role in farmers’ adaptations, but this is rarely examined in the current studies.

### 6.2. Future Areas of Improvement

Linking a robust research approach with local knowledge is important to facilitate adaptation in Africa. With the progress in studying the impacts of weather extremes on crop production, the understanding of the impacts of adaptation to extreme weather events could be improved by targeted studies. International organizations, like CIMMYT, can play important roles in bridging these gaps by integrating local knowledge and new technologies to increase agricultural resilience in Africa. Using the maize cropping system as a base case, we propose several case studies in the following areas to increase the understanding of extreme weather impacts on food systems and the evaluations of adaptation strategies.

- Utilizing datasets from different sources to quantify the disaster risks of extreme weather events tailored to maize production or specific maize-based cropping systems.

Agroclimatic index or risk analyses have been used widely in Africa to investigate the risk of extreme weather events. This analysis must be aligned to the local environment and specific cropping system in greater detail. However, this is usually difficult due to data limitations. In fact, observations and data collection systems in Africa are limited and have been declining for decades. This affects the most basic data, such as weather data, long-term site observation, fine-scale survey or census, and crop distribution data. For instance, estimates of the cropland extent in Africa range from about 1 to more than 6 million km^2^. This scarcity and uncertainty of such basic information add considerable difficulty to the quantification and evaluation of impacts and adaptation options. Some studies have found that what farmers perceive as climate change and extreme weather events have not been confirmed by long-term climate trends [139], suggesting the separation between agroclimatic analysis and consideration of the real on-farm or the heterogeneous local production conditions. There might be technology alternatives that are brought to resolve these data issues: remote sensing and proximal sensing data, breeding trials and on-farm crop cut information, and validation of different land-use products using Wikis and Google Earth. Many of these data can complement land-based observation to enable risk analysis.

- Improving crop models with experimentation and an agroclimatic approach to quantify the impacts of extreme weather events.

Process-based models have proven to be key tools for understanding the processes of the complex interconnections in cropping systems and for assessing climate impacts and adaptation options for crop systems. However, they are not yet fully qualified to adequately capture extreme crop impacts. To achieve further progress, the models can be improved through systematic comparisons and exchanges among empirical statistical and process-based modeling. On one hand, empirical statistical models with attention to various agroclimatic extremes can be used as an alternative to investigate which regions/climatic zones crop models and empirical models agree and disagree. On the other hand, experimentation and empirical databases can be extended to enable the improvement of process-based models to such an extent that they adequately capture the impacts of major agroclimatic extremes and their combinations and deliver robust and accurate estimates on yield penalties. Models should be specially improved for yield reduction mechanisms related to underexamined agroclimatic extremes, such as low rainfall and excessive rainfall, and the yield reduction factors that are linked to climatic extremes, like nitrogen leaching, pest, and disease incidence.

- Combining multiple modeling tools to focus adaptation assessment on resilience rather than maximum productivity.

There is a strong need for successful adaptation across different scales and dimensions of impacts depending upon crops and regions. Models, particularly process-based models, have become the primary approach in Africa to assess adaptation options for increased extreme weather events. Webber et al. [140] reviewed crop model studies in Africa and highlighted two roles that crop models now play. (1) Crop models were used to generate yield response functions for climate and management as inputs to economic models. (2) Crop models were used to analyze interactions of management, environment and climate, organize scientific understanding at a high level of complexity, and to highlight the areas where knowledge gaps exist. Many studies in social sciences report on adaptation in response to some drivers in addition to climate, but no analysis is provided as to how these adapted practices would perform under future climate conditions. In addition, system resilience was highlighted as a successful adaptation goal, as opposed to simply maintaining the highest yield levels. Although many crop models were able to simulate the many bio-physical variables and could be used to substantiate general statements about the ability of adaptation to build resilience, including the factors that affect adaptation and particularly evaluating the efficiency and complications of adaptation, they require the substantial extension of crop models to wider domains, such as water, social, and economic domains. For example, crop models need to be improved to fully integrate with economic or agent-based models if researchers want to fully understand the consequences of the adoption of drought and heat-resistant cultivars. (3) Crop models are required to be combined with hydrological models to investigate adaptation options of large-scale irrigation. Indeed, in addition to crop models, examining system resilience still needs other steps. To do so, emphasis should be placed on defining system boundaries, key variables influencing system resilience, associated indicators, and thresholds that crop models and other sector models can predict. All these require systematic research.

## Data Availability

No new data were created in the study.
